# Factors affecting nutritional knowledge, attitude, practices and dietary intake among national players in Kathmandu, Nepal: a cross-sectional study

**DOI:** 10.1186/s13102-023-00691-7

**Published:** 2023-06-30

**Authors:** Madhu Thapa, Arjun Neupane, Sailendra Kumar Duwal Shrestha, Prabin Nepal, Atul Upadhyaya, Pratik Niraula, Ram Kumar Shrestha, Dev Ram Sunuwar

**Affiliations:** 1 Department of Nutrition and Dietetics, College of Applied Food & Dairy Technology, Purbanchal University, Lalitpur, Nepal; 2 Department of Nutrition and Dietetics, Nepal Armed Police Force Hospital, Kathmandu, Nepal; 3 Maharajgunj Medical Campus, Institute of Medicine, Tribhuvan University, Kathmandu, Nepal; 4 Department of Orthopedic, Nepal Armed Police Force Hospital, Kathmandu, Nepal; 5 Baliyo Nepal Nutrition Initiative, Kathmandu, Nepal; 6 Department of Nutritional Sciences, School of Public Health, University of Michigan, Ann Arbor, USA

**Keywords:** Nutrition knowledge, Attitude, Practice, Dietary intake, National players, Nepal

## Abstract

**Introduction:**

Good nutrition knowledge and nutrient intake have been regarded as significant determinants in enhancing athletes’ performance and overall health status. This study aimed to assess knowledge, attitude, and practices of nutrition and dietary intake among athletes.

**Methods:**

A cross-sectional study was conducted from January to April 2022 among national athletes from two sports clubs in Kathmandu Metropolitan City, Nepal. A semi-structured questionnaire was used to collect the data. Anthropometric measurements and dietary intake were recorded. Bivariate and multivariate binary logistic regression was used to estimate the crude odds ratios (cOR), and adjusted odds ratios (aOR) with 95% confidence intervals (CIs).

**Results:**

A total of 270 players were included in this study (mean age, 25 years; 49.6% male, 50.4% female). Almost half of the athletes had good nutrition knowledge [54.1% (146/270)], attitude [56.7% (153/270)], and practices [50% (135/270)] scores. The mean energy, carbohydrate, protein, and fat intake were 35.0 kcal/kg/day, 5.6, 0.9, and 0.9 g/kg/day, respectively. Likewise, mean calcium and iron intake were 370, and 12.5 mg, respectively. In the multivariate model, families with monthly household income less than 50,000 Nepalese rupees ($400) (adjusted Odds Ratio/aOR = 2.58; 95% CI: 1.12 to 5.96), and those who did not receive diet plan (aOR = 3.14; 95% CI: 1.25 to 7.84) were more likely to have poor nutrition knowledge. Players who did not check food labelling (aOR = 1.44; 95% CI: 0.78 to 2.63) were more likely to have negative attitude towards nutrition. Players who did not ever attend nutrition class (aOR = 3.54; 95% CI: 1.46 to 8.54) and those who did not consume different food during off and on the season of sports (aOR = 2.36; 95% CI: 1.39 to 4.01) were more likely to have poor nutrition practice.

**Conclusions:**

Half of the athletes’ nutritional knowledge, attitudes, and practices score were satisfactory. Nutrient intake among athletes was suboptimal. Nutrition intervention programs are critical to improve nutritional knowledge, attitude and practice related to dietary intake among national athletes in Nepal.

**Supplementary Information:**

The online version contains supplementary material available at 10.1186/s13102-023-00691-7.

## Background

Nutrition plays a significant role to lead a healthy lifestyle [1]. Diet and lifestyle of athletes have an impact on their preparedness for competition, performance, and recovery [2]. Athletes’ nutrition plans should contain provisions for sufficient nutrients to support in recovery as well as preparation for training and competition [3]. Adequate nutrition supports training intensity, muscle recovery, and metabolic adaptations [4]. Nutrition strategies help athletes perform optimally by reducing or delaying the factors that would otherwise cause fatigue [5].

Athletes’ knowledge on nutrition is a factor that can affect their eating habit [3]. Athletes who have a good understanding of nutrition can recognize the need of training and perform at their best [6]. Also, athletes with higher nutrition knowledge were more likely to consume more fruit, vegetables, and carbohydrate-rich foods than those with low nutrition knowledge [7]. The association between nutrition knowledge and dietary behavior is multifaceted and influenced by many other individual and environmental factors such as hunger, appetite, taste, food preferences, beliefs, culture, and experiences [8–10]. When one has a complete understanding of nutrition, they tend to develop positive attitude towards nutrition. Positive nutrition attitude have been linked to better diets, promoting healthier food (higher healthy eating index), and nutrient dense foods in proper amount [11]. Understanding the various nutrition related practice of athletes is important, as it influence energy consumption, nutrient intake, and hydration status [12]. There are several recommendations related to nutrition suggested for athletes to boost their physical performance and maintain health by several organizations [13–15]. However, demanding training and travel schedules without proper nutritional knowledge can impair them from optimal dietary intake [16]. Understanding the nutritional habits of athletes can help plan and implement appropriate nutrition education ultimately enhancing the knowledge and potentially influencing the nutrition practices of athletes [17].

Athletes are in need of adequate macro & micronutrients, and fluids including meal timing [15]. For instances, meeting energy demand and maintenance of body mass and body fat at appropriate levels are key goals of nutrition [18], and are linked to poor knowledge on nutrition information which could ultimately lead to unhealthy eating practices [6, 19, 20].

Inadequate nutrition is also a key determinant for the high burden of injury among athletes [21]. Most commonly observed was inadequate energy intake relative to exercise energy expenditure, which led to Relative Energy Deficiency in Sports (RED-S) [22]. The International Olympic Consensus expert working group has introduced a broader, more comprehensive term for the condition previously known as ‘Female Athlete Triad’ [23]. Female athlete triad stands for energy availability, menstrual function and bone health [22]. Today, it is not limited among females and observed among males as well [24]. The syndrome of RED-S is defined as “impairments of metabolic rate, menstrual function, bone health, immunity, protein synthesis, cardiovascular health, and other physiological functions, caused by relative energy deficiency” [23].

Limited number of studies were undertaken so far in sports nutrition in Nepal. A previous study conducted in Nepal investigated the association of nutritional knowledge, practice, supplement use, and nutrient intake with strength performance, however the study only looked at Taekwondo players [25]. There is a lack of research on the understanding of nutrition, nutritional status, dietary intake, and sources of information among national-level athletes in Nepal. Despite studies conducted on the general population, this specific group (athletes) has not been studied yet. This study is designed to explore gaps and areas of knowledge deficits to inform the intervention. The main objective of this study was to assess nutrition knowledge, attitude, practices, and dietary intake among athletes and their interrelation.

## Methods

### Study design and setting

A cross-sectional study was conducted among 270 athletes from January 18, 2022, to April 10, 2022. Nepal Armed Police Force (APF) club and the Nepal Police club were selected purposively because both clubs are affiliated to Government of Nepal with a variety of sports teams and represent national level athletes. Male and female athletes between the ages of 18 and 40 were included in this study. Players suffering from severe injury, illness, players who were not present on the day of data collection, and players who were not willing to participate were excluded from the study.

### Sample size, sampling strategy

A total sample size of 290 was estimated based on the single proportional formula n = Z^2^pq/d^2^; taking 50% proportion rate with α level of significance at 5%. In the formula, Z = standard normal deviation and equaled 1.96 at α level of significance; p is the prevalence of the outcome of interest which was set at 0.5 considering the unknown prevalence of nutritional knowledge or nutritional practice among national players in the study area; q = 1-p; and the margin error (d) was set at 6% and 10% non-response rate was added.

Convenience sampling was used to select the sports club. First, the Nepal APF Club and the Nepal Police club were selected purposively. After that list of total players from each club were retained by coordinating with authority of respective clubs. Consecutive sampling technique was used to collect data from all the available list of players, where the players were interviewed, and data was collected until the desired sample size was met.

### Data collection tools and technique

A face-to-face interview was conducted using a semi-structured questionnaire. Socio demographic information, behavioral information, anthropometric measurement, nutrition knowledge, attitude, and practices information were collected. Socio demographic questionnaire were prepared based on the previous literature and revised appropriately for Nepalese context [25, 26]. Nutrition knowledge, attitude, practice (KAP) score questionnaires were developed based on the previously validated tools [25–28]. We approached the athletes in the morning for the data collection, as the training session was held in the morning every day, and we had the opportunity to meet the majority of them simultaneously. We collected participant’s anthropometric measurements, and information on dietary intake of athletes.

Data collection tool was developed in English language and then translated into the Nepali language and back-translated into the English language to ensure the validity of the questionnaire. Pretesting of the tool was carried among 30 players from another private club. Interviews were conducted at the respective club premises and each interview lasted for up to 40 minutes. Two trained enumerators who were studying Master of Science in Nutrition and Dietetics in their final year conducted the interviews. A field supervisor confirmed the quality of the data by cross verifying the completed questionnaire on-site, and any discrepancies were discussed with enumerators.

### Study variables

#### Predictor variables

Information on participant’s age, sex, ethnicity, religion, education, monthly income, sources of information, duration of involvement in sports, sports category, daily training, previous training/classes history, and supplements use were collected. For age, two categories were made 18 to 24 years and above 24 years. Ethnicity was classified as *Brahmin/Chhetri*, *Janajati*, *Dalit, Madhesi* caste and others. For further analysis of education qualifications, we coded up to 10 class as “Secondary level and below” class 11–12 was coded as “Higher secondary” and the rest was coded as “Bachelor’s degree and above” Sports category was prepared referring to guidelines developed by International Life Sciences Institute India, National Institute of Nutrition and Sports Authority India [29]. Educational qualification was coded as secondary level and below, higher secondary and bachelor’s and above. Monthly family income was further categorized as below 50,000 Nepalese rupees (NRs) ($400) and NRs 50,000 and above. Multiple response questions were included for sources of information related to nutrition, options to the question were books, articles, social media, coach/trainers, friends, newspaper, medical personnel, dietitian/nutritionist and others. The daily training period was categorized as less than three hours and more than three hours.

Height, weight, and body mass index (BMI) were calculated according to WHO classification [30]. The anthropometric measurements including the weight and height of the participants were measured according to standardized procedures. Weight was measured to the nearest 0.1 kg using an Omron digital weighing. Height was measured in the standing position to the nearest 0.1 cm with a Seca 213 portable stadiometer. The BMI was calculated using weight (kg) divided by height squared (m^2^) and categorized using the WHO [30]. Body composition measurement such as body fat (BF), visceral fat level (VFL), and skeletal muscle (SM) were measured using Bioelectrical Impedance Analysis (BIA). The BIA is a widely available, low-cost and non-X-ray-based method, and is used frequently to evaluate body composition [31]. All participant’s body fat, visceral fat and skeletal muscle were measured in lightweight clothing and standing barefoot on the metal foot pads. To measure the bio-impedance, a very low, safe electrical signal was sent from four metal electrodes through the feet to the legs and abdomen. Participant’s information were recorded into the system to enable the calculating of the BIA algorithms, included gender, age, height and weight. Body fat percentage, visceral fat level, and skeletal muscle were recorded [32].

We used a pre-tested 24-hour dietary recall interview intended to capture detailed information about all foods and beverages consumed by the athletes in the past 24 h (from midnight to midnight the previous day). We enrolled all the athletes during their training time for the data collection. During 24-hour recall, the athletes were asked to name all the food and drink items consumed during the preceding day, including anything consumed outside the home and the time of consumption was also recorded. If multiple servings of the same food items were reported to be consumed in a single eating occasion, then these amounts were combined to a single portion. The portion size of items consumed was estimated using graduated measuring cylinder and standard weight for foods that are served as a unit (boiled egg, bread slice), as per the principle and guideline of the Indian Institute for Medical Research (ICMR). Based on the information obtained from the 24-hour dietary recall method, the amount of food was then converted into daily nutrient intakes. The mean daily intake of total calorie, carbohydrate, protein, fats, calcium, and iron over 24 h recalls was calculated accordingly using the Nutrition Society of India (NSI) diet calculator developed by the National Institute of Nutrition, Indian Council for Medical Research, Hyderabad, India. Along with it, for the items that were not listed in NSI diet calculator, food composition table of Nepal was used [33]. The mean daily energy, protein, carbohydrate, and fat intake was compared with the values reported in the Nutrition and Hydration Guidelines for Excellence in Sports Performance, National Institute of Nutrition, India [29] and current American College of Sports Medicine (ACSM) sports nutrition guidelines [5].

#### Outcome variables

Knowledge, attitude, practice (KAP) score questionnaire were adapted from previously published literature [25, 26]. The knowledge section had 28 statements which could be answered as ‘Yes’, ‘No’ and “don’t know”. Each correct response was coded as ‘1’ and the incorrect one as ‘0’. In the attitude section, there were a total of 16 statements, which could be answered on Likert scale ranging from “strongly agree” to “strongly disagree”. The scores ranged from “0–2”, where ‘2’ was the most positive and ‘0’ was the most negative. In the practice section, 14 statements were prepared with their response as ‘yes’ or ‘no’. Each positive response in this section was scored as ‘1’ and negative responses were scored as ‘0’. After scoring, the score for all of the answered questions for knowledge, attitude and practice was summed up separately. The higher score meant participants have good knowledge, practice, and positive attitude towards nutrition. To decide the individual as having poor or good knowledge score and practices and positive or negative attitude towards nutrition, the median score was used as the cutoff point since scores were not normally distributed. The questionnaire on nutrition knowledge, attitude, and practice have Cronbach alpha values of 0.80, 0.75, and 0.60, respectively.

### Data management and analysis

Data checking and compiling was done manually to ensure completeness and accuracy before data were entered for analysis. The collected data were entered into EpiData software 3.1v and transferred into Stata/MP version 14.1 (StataCorp LP, College Station, Texas) for statistical analysis. The normality of the distribution of continuous variables was evaluated using the Shapiro wilk test. The descriptive results were presented in the form of mean, standard deviation, frequency, and percentage for normally distributed data and non-normally distributed data were expressed as median and interquartile range. Descriptive results were presented for nutrient intake.

Pearson’s chi-squared (χ2) test was applied for examining associations of categorical variables with knowledge, attitude, and practice score. Moreover, variables that lead to good or poor KAP scores were analyzed using bivariate and multivariate binary logistic regression. Results were presented as crude odds ratio (cOR) and adjusted odds ratio (aOR) with 95% confidence intervals (CIs). Associations with a p ≤ 0.20 in the bivariate analyses were included in the multivariate logistic regression models. P-values < 0.05 were considered statistically significant.

### Ethical considerations

All methods of this study were carried out under the Declaration of Helsinki’s ethical principle for medical research involving human subjects [34]. The ethical clearance for this study was obtained from the ethical review board of the Nepal Health Research Council (NHRC) (Reference number: 1829/2021). Formal permission was also obtained from the respective Clubs. The written informed consent was obtained from all eligible participants before proceeding with the data collection. Also, the data enumerators elaborated the objectives of the study among each athlete, and they were informed about voluntary participation, their right to refuse at any point, and the confidentiality of their identity.

## Results

### Socio demographic characteristics

Of 290 participants, a total of 270 participants (male: 49.6%, and female: 50.4%) completed the study and the response rate was 93.1%. The mean (SD) age of players was 25.8 (4.2) years. More than half of respondents were *Janajati* (55.5%) followed by *Brahmin/Chhetri* (36.6%) ethnicity. Nutritional Assessment showed that most of the players (79.6%) had normal BMI, whereas 18% were overweight/obese. About body composition, the mean body fat, skeletal muscle and visceral fat of players were 25.7%, 30.4% and 5.4%, respectively. Half of respondents (50.3%) had an educational qualifications of higher secondary followed by secondary level (40.3%), and bachelor’s level and above (9.2%). Most of participants (79%) were playing team event sports such as football, cricket, volleyball, and Kabaddi, whereas 11% were reported from power events like weight lifting and 7% from light events like gymnastic. More than two-thirds of players (69.6%) reported performing less than three hours of training each day (Table 1).

**Table 1:** Demographic and anthropometric characteristics among athletes (n = 270)

Characteristics	Frequency	Percentage
**Age, mean** **±** **SD**	25.8 ± 4.2	
**Age**		
18–24 years	121	44.8
25–39 years	149	55.1
**Gender**		
Male	134	49.6
Female	146	50.3
**Ethnicity**		
Brahmin/Chhetri	99	36.6
Janajati	150	55.5
Dalit	13	4.8
Terai caste	4	1.4
Others	4	1.4
**Monthly Family Income (1USD = 125 Nepalese rupees)**		
Rs 50,000 and below	232	85.9
Above Rs 50,000	38	14.1
**Education level**		
Secondary level and below	109	40.3
Higher secondary level	136	50.3
Bachelor level or above	25	9.2
**Department of sports**		
Power event	27	10
Endurance event	21	7.78
Light event	4	1.48
Team events	218	80.70
**Marital status**		
Unmarried	97	72.96
Married	73	27.04
**Club Affiliation**		
Nepal police club	85	31.48
Nepal APF club	185	68.52
**Daily duration of training**		
3 hours and less	188	69.6
More than 3 hours	82	30.3
**BMI, mean** **±** **SD**	23.1 ± 2.8	
**BMI Classification**		
Underweight	6	2.2
Normal	215	79.6
Overweight & Obese	49	18.0
**Body fat percentage, mean** **±** **SD**	25.8 ± 4.7	
**Skeletal muscle mass, mean** **±** **SD**	30.5 ± 2.7	
**Visceral fat percentage, mean** **±** **SD**	5.4 ± 3.1	

### Nutrition related information

Only 7.4% of athletes reported using dietary supplements. Also, most of them use whey protein supplement (75%) followed by multivitamin (25%). About 14% of participants have attended nutrition related class. More than half (55%) of players did not have any difference in food intake during the off and on season of sports event. In addition, almost 13% had been following diet plans and among them the source of plan was coach, followed by medical personnel, friends and social media. More than one-third (37%) reported checking food labelling before purchasing or consuming any food items, whereas 63% did not check the food labelling (Table 2).

**Table 2:** Nutrition related information among athletes (n = 270)

Characteristics	Frequency	Percentage
**Dietary supplements use**		
Yes	20	7.4
No	250	92.6
**Type of dietary supplement use**		
Protein powder	15	75
Multivitamin	5	25
**Nutrition class ever attended**		
Yes	38	14.0
No	232	85.9
**Is there any difference in food you eat during off and on season?**		
Yes	117	43.3
No	153	56.6
**Do you follow any diet plan?**		
Yes	35	12.9
No	235	87.0
**Read Food labelling before purchasing food**		
Yes	100	37.0
No	170	62.9
**Nutrition knowledge score, mean** **±** **SD (**Minimum − Maximum)	19 ± 4.8 (4 − 28)	
**Nutrition knowledge**		
Good	146	54.1
Poor	124	45.9
**Attitude score, mean** **±** **SD (**Minimum − Maximum)	11.5 ± 1.6 (7 − 15)	
**Attitude toward nutrition**		
Good	153	56.7
Poor	117	43.3
**Practice score, mean** **±** **SD (**Minimum − Maximum)	7.4 ± 2.1 (1 − 12)	
**Practice**		
Good	135	50.0
Poor	135	50.0

The mean (SD) knowledge, attitude and practice score of participants were 19 (4.8) 11.5 (1.6), and 7.4 (2.1), respectively. More than half of the athletes had good nutrition knowledge [54.1% (146/270)], and attitude [56.7% (153/270)] scores, whereas half of players had practice [50% (135/270)] score (Table 2).

**Fig. 1:**
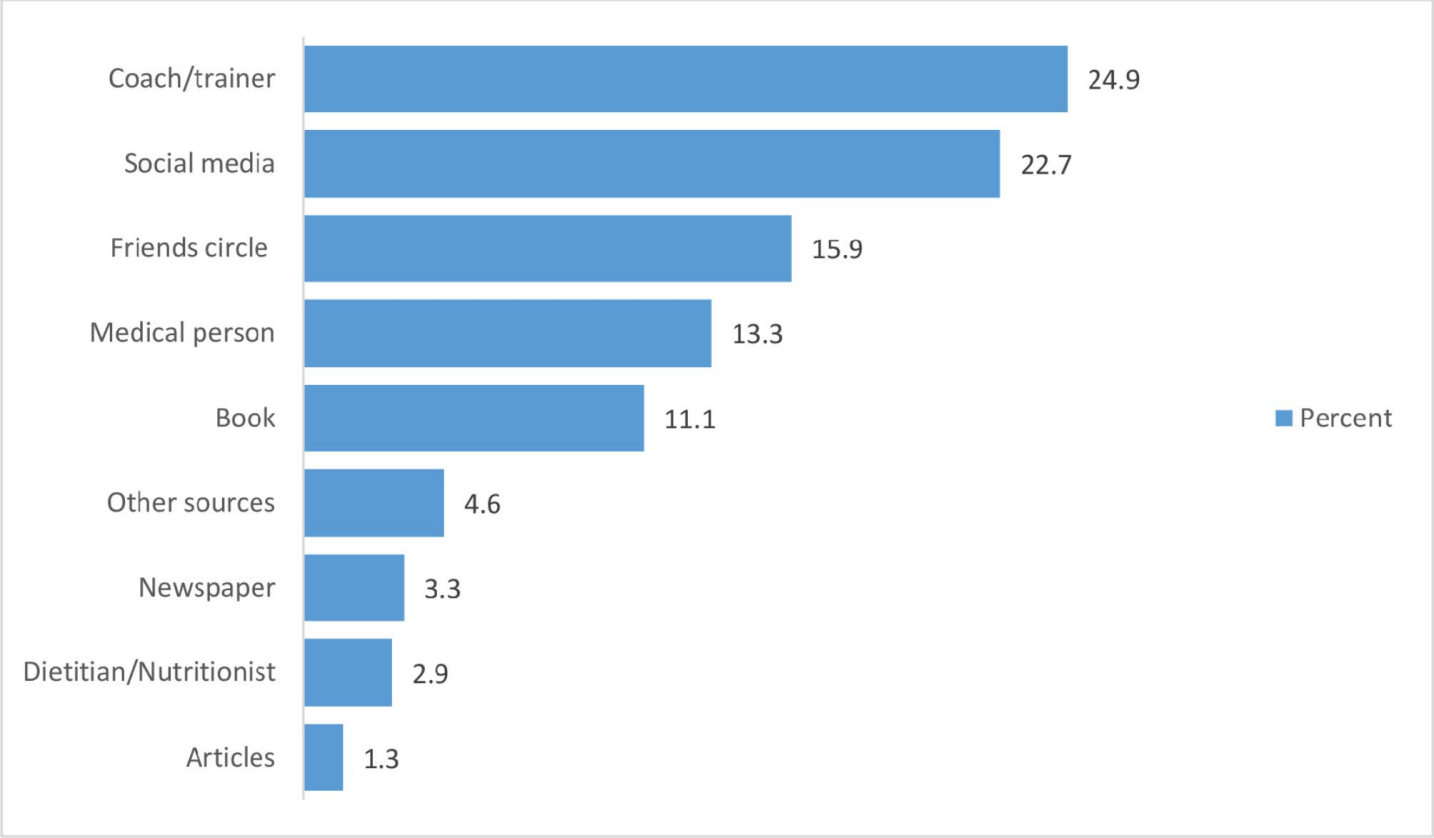
Sources of information regarding nutrition among players

In this study, athletes relied more on coaches/trainers (24.9%), followed by social media (22.7%), friend circle (15.9%), medical professionals (13.3%) and books/newspaper/article (15%), whilst only 3% reported dietitian/nutritionist for nutrition related information (Fig. 1).

### Nutrient intake among athletes

The median (IQR) energy intake was 2200 (1450) kcal. Median (IQR) protein intake relative to body weight per day was 0.9 (1)g and the fat intake was 0.9 (0.9)g relative to body weight per day (44.1)g. Similarly, median carbohydrate intake per kg body weight per day was 5.6 g. The median (IQR) percentage of carbohydrate, protein, and fat intake of the participants were 66% (17.1), 11.1% (8.9), and 22.6% (16.1), respectively. With regard to micronutrient intake, median calcium intake was 370 mg. Similarly, median iron intake was 12.5 mg.

Among the four sports category, median (IQR) energy intake was highest among team events, while least intake among power events. Related to protein intake, light event-players had highest intake, whereas intake was less among endurance event-players. Carbohydrate intake was highest among endurance event athletes and less intake were found among power events players. Power event-players had highest fat intake and endurance event-players had lowest fat intake (Table 3).

**Table 3:** Nutrient intakes among athletes (n = 270)

Nutrients	Total players	Male Players	Female Players	Power events Players	Endurance events Players	Light events Players	Team events Players
Energy (Kcal), Median (IQR)	2200 (1450)	2290 (1400)	2132 (1050)	2100 (780)	2150 (157)	2115 (360)	2200 (1450)
Energy, kcal.kg ^− 1^.d^− 1^Median IQR	35.0 (23.2)	33.5 (21.7)	36.6 (22.0)	33.2 (15.4)	35(8.2)	30.5 (18.0)	35.4 (23.4)
Protein, g.kg^− 1^. d^− 1^ Protein, %	0.9 (1.0) 11.1 (8.9)	0.9 (0.8) 11.1 (6.9)	1.0 (0.9) 11.1 (8.2)	0.9 (0.5) 11 (3.8)	0.9 (0.2) 10.6 (1.3)	0.9 (0.8) 12.1 (4.3)	0.9 (1.0) 11.2 (8.9)
Carbohydrate, g.kg^− 1^. d^− 1^ Carbohydrate %	5.6 (4.5) 66 (17.1)	5.5 (3.9) 66.5 (22.1)	5.9 (4.3) 65.2 (21.4)	5.2 (2.7) 64.6 (12.5)	5.8 (1.4) 68.6 (6.6)	5.1 (1.3) 65.7 (12.1)	5.7 (4.5) 65.8 (22.3)
Fat, g.kg^− 1^. d^− 1^ Fat, %	0.9 (0.9) 22.6 (16.1)	0.8 (0.8) 22.2 (13.9)	0.9 (0.8) 23.4 (14.0)	0.9 (0.5) 23.8 (10.8)	0.7 (0.2) 20.5 (5.4)	0.73 (0.7) 22.1 (7.9)	0.9 (0.9) 23 (14.4)
Calcium (mg), Median (IQR)	370 (260)	410 (561.5)	342 (557.4)	334(210)	320 (70)	320 (346.4)	400 (660.5)
Iron (mg), Median (IQR)	12.5 (11)	13.1 (13.9)	12 (15.5)	11.3 (9.2)	12.1 (4)	9.6 (13.2)	12.7 (15.9)

### Nutritional knowledge attitude and practice

Older players (25–39 years) were shown to have good nutrition knowledge score. Both male and female had similar knowledge score. Among all, higher educational level players had better knowledge score than others. Players with more than Rs 50,000 ($400) monthly family income were having good knowledge score than those below Rs 50,000. Players who had attended nutrition class before had good knowledge score than those who reported not attending any classes before. Players who did not follow any diet plan had poor knowledge score than those who followed the plan. Also, players who checked food labelling were shown to have good knowledge than those who did not check. Players aged above 25 years had a positive nutrition attitude than below 25 years. Higher educational level players had a positive nutrition attitude than other educational qualifications. Players who checked food labelling had positive nutrition attitude than others. For practice score, players with secondary level educational qualification had better nutrition practice score than another educational group. Players with monthly family income above Rs 50,000 were having good practice score than those with low monthly income. Players who did not follow diet plan had poor nutrition practice score than those who followed. Also, Players who had different food intake on and off season had good nutrition practice score than those who had no difference. Also, those who checked food labelling had good practice score than those who did not check it (Table 4).

**Table 4:** Association between baseline characteristics and nutrition related information with nutrition knowledge, attitude and practice

Variables	Knowledge			Attitude			Practices		
	Poor n (%)	Good n (%)	P value^1^	Negative n (%)	Positive^1^ n (%)	P value^1^	Poor n (%)	Good n (%)	P value^1^
**Age**									
18–24	54 (43.5%)	67 (45.8%)	0.700	52 (44.4%)	69 (45.1%)	0.915	60 (44.4%)	61 (45.1%)	0.903
25–39	70 (56.4%)	79 (54.1%)		65 (55.6%)	84 (54.9%)		75 (55.5%)	74 (54.8%)	
**Gender**									
Male	59 (47.5%)	75 (51.3%)	0.535	57 (48.7%)	77 (50.3%)	0.793	68 (50.3%)	66 (48.8%)	0.808
Female	65 (52.4%)	71 (48.6%)		60 (51.2%)	76 (49.6%)		67 (49.6%)	69 (51.3%)	
**Education Classification**									
Secondary level and below	52 (41.9%)	57 (39%)	0.779	56 (47.8%)	53 (34.6%)	0.230	53 (39.2%)	56 (49.4%)	0.460
Higher secondary level	62 (50%)	74 (50.6%)		53 (45.3%)	83 (54.2%)		72 (53.3%)	64 (47.4%)	
Bachelor’s and above	10 (8%)	15 (10.2%)		8 (6.84%)	17 (11.4%)		10 (7.4%)	15 (11.1%)	
**Monthly Family Income (1USD = 125 Nepalese rupees)**									
Rs 50,000 and below	114 (91.0%)	118 (80.8%)	0.009*	105 (89.7%)	127 (83.0%)	0.115	119 (88.1%)	113 (83.7%)	0.294
Above Rs 50,000	10 (8.0%)	28 (19.1%)		12 (10.2%)	26 (16.9%)		16 (11.8%)	22 (16.9%)	
**Clubs Affiliation**									
Nepal Police club	39 (31.4%)	46 (31.5%)	0.992	36 (30.7%)	49 (32.0%)	0.826	39 (28.8%)	46 (34.0%)	0.359
Nepal APF club	85 (68.5%)	100 (68.4%)		81 (69.2%)	104 (67.9%)		96 (71.1%)	89 (65.9%)	
**Nutrition class attended**									
Yes	11 (8.8%)	27 (18.4%)	0.023*	12 (10.2%)	26 (16.9%)	0.115	8 (5.3%)	30 (22.2%)	< 0.01*
No	113 (91.1%)	119(81.1%)		105 (89.7%)	127 (83.1%)		127 (94%)	105 (77.7%)	
**Diet Plan**									
Yes	28 (19.1)	7 (5.6)	0.001*	11 (9.4%)	24 (15.6%)	0.128	12 (8.8%)	23 (17.0%)	0.046
No	118 (80.8)	117 (94.3)		106 (90.6%)	129 (84.3%)		123 (91.1%)	112 (82.9%)	
**Difference in Food intake off and on season**									
Yes	43 (34.6%)	74 (50.6%)	0.008*	45 (38.4%)	72 (47.0%)	0.158	43 (31.8%)	74 (54.8%)	< 0.01*
No	81 (65.3%)	72 (49.3%)		72 (61.5%)	81 (52.9%)		92 (68.1%)	61 (45.1%)	
**Checking Food Labelling**									
Yes	87 (70.1%)	118 (80.8%)	0.041*	78 (66.6%)	127 (83.0%)	0.002*	100 (74.0%)	105 (77.1%)	0.477
No	37 (29.8%)	28 (18.1%)		39 (33.3%)	26 (16.9%)		35 (25.9%)	30 (22.2%)	

^1^ chi square test; *statistically significant at p < 0.05

### Association of determining factors with nutritional knowledge, attitude and practice among athletes

Bivariate analysis showed that athletes having monthly family income Rs 50,000 and below ($400) (crude Odds Ratio/cOR = 2.7, 95% CI: 1.25 to 5.82) and athletes who did not attend nutrition class before (cOR = 2.33, 95% CI: 1.10 to 4.91) were more likely to have poor nutrition knowledge score. Also, those who did not check food labelling (cOR = 2.44, 95% CI: 1.38 to 4.32) had higher odds of having negative attitude towards nutrition. Players who had never attended nutrition class (cOR = 4.53, 95% CI: 1.99 to 10.31) and those who didn’t had difference in food intake during off and on season (cOR = 2.5, 95% CI: 1.58 to 4.26) were more likely to have poor nutrition practice score (Table 5).

**Table 5:** Bivariate and Multivariate logistic regression analysis for determining factors of nutrition knowledge, attitude, and practice among athletes

	Knowledge		Attitude		Practice	
	Bivariate cOR (95% CI)	Multivariate aOR (95% CI)	Bivariate cOR (95% CI)	Multivariate aOR (95% CI)	Bivariate cOR (95% CI)	Multivariate aOR (95% CI)
**Age**						
18–24	1.09 (0.67 to1.77)	0. 91 (0.53 to 1.56)	0.97 (0.60 to1.58)	1.02 (0.60 to 1.71)	0.97 (0.60 to 1.56)	0.96 (0.56 to 1.66)
25–39	Ref	Ref	Ref	Ref	Ref	Ref
**Gender**						
Male	Ref	Ref	Ref	Ref	Ref	Ref
Female	1.16 (0.72 to 1.87)	1.18 (0.70 to 1.97)	1.06 (0.65 to1.72)	1.02 (0.61 to1.77)	0.94 (0.58–1.51)	1.03 (0.61 to 1.72)
**Education Classification**						
Secondary level and below	1.36 (0.56 to 3.32)	0.92 (0.33 to 2.51)	2.24 (0.89 to 5.63)	2.02 (0.73 to 5.5)	1.41 (0.58 to 3.43)	1.22 (0.45 to 3.34)
Higher secondary level	1.25 (0.52 to 2.99)	0.84 (0.32 to 2.20)	1.35 (0.54 to 3.36)	1.18 (0.44 to 3.14)	1.68 (0.70 to 4.02)	1.41 (0.54 to 3.70)
Bachelor’s and above	Ref	Ref	Ref	Ref	Ref	Ref
**Monthly Family Income (1 USD = 125 NRs)**						
Rs 50,000 and below	2.7 (1.25 to 5.82) *	2.58 (1.12 to 5.96) *	1.79 (0.86 to 3.72)	1.49 (0.67 to 3.28)	1.44 (0.72 to 2.89)	1.22 (0.56 to 2.65)
Above Rs 50,000	Ref	Ref	Ref	Ref	Ref	Ref
**Clubs Affiliation**						
Nepal Police club	Ref	Ref	Ref	Ref	Ref	Ref
Nepal APF club	1.00 (0.59 to 1.67)	1.11 (0.61 to 2.01)	1.06 (0.63 to 1.78)	1.10 (0.61 to 1.99)	1.27 (0.76 to 2.12)	1.25 (0.68 to 2.27)
**Nutrition class attended**						
Yes	Ref	Ref	Ref	Ref	Ref	Ref
No	2.33 (1.10 to 4.91)*	1.5 (0.65 to 3.48)	1.79 (0.86 to 3.72)	1.19 (0.53 to 2.66)	4.53 (1.99 to 10.31)*	3.54 (1.46 to 8.54)*
**Diet Plan**						
Yes	Ref	Ref	Ref	Ref	Ref	Ref
No	3.96 (1.66 to 9.43)*	3.14 (1.25 to 7.84)*	1.79 (0.83 to 3.82)	1.47 (0.65 to 3.33)	2.1 (1.00 to 4.42)*	1.40 (0.61to 3.17)
**Difference in Food intake off and on season**						
Yes	Ref	Ref	Ref	Ref	Ref	Ref
No	1.93 (1.18 to 3.16)	1.61 (0.96 to 2.74)	1.42 (0.87 to 2.32)	1.28 (0.75 to 2.17)	2.5 (1.58 to 4.26)*	2.36 (1.39 to 4.01)*
**Reading Food Labelling**						
Yes	Ref	Ref	Ref	Ref	Ref	Ref
No	1.79 (1.02 to 3.14)	1.44 (0.78 to 2.63)	2.44 (1.38 to 4.32)*	1.44 (0.78 to 2.63)*	1.22 (0.70 to 2.14)	0.87 (0.47 to 1.60)

Ref: reference category; Binary logistic regression was used to calculate crude and adjusted odds ratios; cOR = crude odds ratio; aOR = adjusted odds ratio; Variables p ≤ 0.20 from the bivariate analysis were adjusted in the multivariate model; *statistically significant at p < 0.05.

Multivariate logistic regression models indicated that athletes having monthly family income Rs 50,000 and below ($400) (adjusted Odds Ratio/aOR = 2.5, 95% CI: 1.12 to 5.96) and athletes not following diet plan compared to those following diet plan (aOR = 3.14, 95% CI: 1.25 to 7.84) were more likely to have poor nutrition knowledge score. Similarly, those who did not check food labelling (aOR = 1.44, 95% CI: 0.78 to 2.63) had higher odds of negative attitudes towards nutrition. Also, athletes who had never attended nutrition class (aOR = 3.54, 95% CI: 1.46 to 8.54) and those who did not have difference in food intake during off and on season (aOR = 2.36, 95% CI: 1.39 to 4.09) were more likely to have poor diet practice score (Table 5).

## Discussion

This study adds to the paucity of knowledge, attitude and practice related to athletes in Nepal. This is the first study to explore nutrition knowledge, attitude, practices, and dietary intake among players from a variety of sports. The findings of this study showed a half of the athletes had good knowledge, attitude, and practice related to nutrition. Total energy intake, protein, and fat intake were low according to the current recommendations for athletes.

In this study, more than half of respondents had good nutrition knowledge score which is consistent with the findings of previous study conducted in Bangladesh [26]. This may be due to the similar environmental and socioeconomic background of players. However, in contrast to a prior study by Sunuwar et al. (2022) among Taekwondo players in Nepal, less than half of players had good nutrition knowledge and practice scores [25]. Another study among Rugby players also revealed contrasting result [35]. In our study, there was no significant difference in knowledge score among male and female athletes and similar findings were observed in other studies [36, 37]. Study conducted among sports trainee of Bangladesh showed significant association of gender with nutrition knowledge score, with males likely to have good nutrition knowledge score than females [26]. Unadjusted model showed that those players who did not attend previous nutrition class were more likely to have poor nutrition knowledge.

Nutrition training can offer valuable information about healthy diet, nutrients, and various events’ meal necessary for the players. The primary source of information for nutrition were coaches and social media. Very few of them sought nutrition advice from a nutritionist/dietician. The result was consistent with another study, where most athletes preferred athletic trainers/coaches to dietitians to access nutrition information [38]. Few research were conducted to assess nutrition knowledge among coaches & athletic trainers and it showed inadequate knowledge among them [37, 39]. Another study revealed that athletes perceived athletic trainers to have adequate nutrition knowledge [37]. Moreover, there is lack of nutrition professional in the sports institution who can provide evidence-based nutrition knowledge even in developed nation, while institutions Nepal are unaware of the nutritionist/dietitian. To provide guidance to athletes, coaches and trainers with adequate authentic nutrition information are necessary [38].

Monthly family income and diet plan adherence was found significantly associated with nutrition knowledge score. Those players who had low income may not get access to relevant information source leaving them with poor knowledge. Those following diet plan may tend to inquire more about nutrition information and learn more than their counterparts. Regarding attitude, more than half respondents had shown positive attitude towards nutrition similar to recent study [26]. Nutrition knowledge has significant association with attitude and this finding was supported by previous study [40]. The prevalence of supplement use among players was 7.8% which was similar with recent study conducted among Taekwondo players in Nepal [25]. Whereas, use of supplements was found to be higher in other studies conducted in Egypt [41] and Turkey [42]. A study revealed that most common reasons for supplement use among athletes include a belief that the stress of intense training/competition cannot be met by food alone, and that supplements can offer a specific advantage in either training or competition [43].

Food labelling checking was associated with attitude score. Players who checked the food labelling tended to have positive attitude towards nutrition. Previous exposure to nutrition class/lecture also showed significant association with practices score and nutrition knowledge among athletes [44]. Nutrition intervention also led to positive impact on dietary habit among adolescents, where they increased the frequency of meal and decreased the meal timing and focused on pre and post training meals [45] .

In the study, a total energy intake, protein, fat, calcium, and iron were significantly lower than the corresponding recommended dietary allowances. No athletes met the recommendations for energy intake in this study. Similar findings were observed in the previous study conducted among Australian football athletes and cross fit players [46, 47]. This may be due to hectic training schedule, travels, lack of adequate knowledge about diet quantity, and quality among them. An adequate energy intake supports optimal body function, determines the capacity for intake of macronutrients and micronutrients, and assists in manipulating body composition in athlete [48].

With related carbohydrate intake, all athletes met carbohydrate recommendations which is consistent with the previous study [25]. Carbohydrates are the main source of energy in athletes’ diets. Athletes require an adequate intake of carbohydrates in order to build up muscle glycogen reserves and thus provide the necessary energy for muscle work [47]. The median carbohydrate intake for endurance was in line with the the recommendations suggested by International life sciences Institute, National Institute of Nutrition and Sport Authority of India [29]. This result is consistent with results from Sunuwar et al. (2022) [25] and Ali A et al. (2015) [49].

The median fat intake of athletes was 22.6% with individual median intake of endurance, light events and team events 20.5%, 23.4% and 22.5%, respectively. The median intake of calcium and iron for athletes was 370 mg and 12.5 mg, respectively. Majority of athletes fails to meet the RDA of Calcium (1000 mg). These findings are consistent with previous studies [7, 25] which showed inadequate intake of calcium and iron. Adequate calcium is necessary throughout the life to optimize bone health which if compromised, can lead to bone stress injuries and early osteoporosis [50–52]. Iron is crucial mineral used by the body for numerous processes such as oxygen transport and energy production at a cellular level. Intake of iron by study participants was similar to the findings from the cross sectional study conducted by Pate et al. (2022) [53]. Also, the mean body fat, skeletal muscle, and visceral fat of players were 25.7%, 30.4% and 5.4%, respectively, which is higher than previous studies [54, 55]. This could be due to inter population variability of body compositions and tools used for measurement.

Our study had a few limitations. 24-hour dietary recall was used to calculate dietary intake which may have incurred recall bias. We only included calcium and iron as micronutrient intake, despite the fact that additional minerals and vitamins are crucial to human health and nutritional status. Since the information was collected through face-to-face using a structured questionnaire, response preference to some nutrition issues may have led to social desirability bias. Similarly, data collection timing may have affected the results as some athletes were on preparatory phase and some on competition phase. Similarly, for body composition assessment, we used Bioelectrical Impedance Analysis (BIA), which may overestimate the body fat percentage. Despite the above noted shortcomings, our study has several strengths. This study examined a wide range of sports categories that included national athletes and reflected overall nutritional knowledge, attitudes, practices, and nutrient intake among athletes in Nepal. The association between nutritional knowledge, attitude, practice, and many variables was thoroughly analyzed in our study. The findings of this study can be used as a guideline to enhance the dietary guidelines for all kinds of players in Nepalese context.

## Conclusions

Half of the players had good nutrition knowledge, practice and attitude scores. Dietary intake among players were below the recommendations of sports guidelines. Family income and who followed diet plan were found to have good nutrition knowledge score. Those athletes who attended nutrition class, and consumed food according to off and on season was found significantly associated with good practice score. More studies with wider population are needed to identify robust relationship of various factors with KAP and dietary intake. Also, Study focusing among coaches and trainers can be carried out to know the level of nutrition knowledge among them.

## Electronic supplementary material

Below is the link to the electronic supplementary material.

Supplementary Material 1

## Data Availability

Dataset used in this study is available upon reasonable request to the corresponding author.
