# Supplementary material for: Factors affecting nutritional knowledge, attitude, practices and dietary intake among national players in Kathmandu, Nepal: a cross-sectional study
Source: BMC Sports Sci Med Rehabil. 2023 Jun 30;15:73. doi: 10.1186/s13102-023-00691-7 (PMC10311800; doi:10.1186/s13102-023-00691-7)
Supplement: Supplementary file 1 — Supplementary Material 1 [file 13102_2023_691_MOESM1_ESM.pdf]

Factors affecting nutritional knowledge, attitude, practices and dietary intake among national players in Kathmandu, Nepal: a cross-sectional study

Questionnaire on Research Among players about sports nutrition		
**SN**	**Questions**	**Details**
1.	Date	
2.	Name:	
3.	Gender	☐ female<br>☐ male
4.	years	
5.	Height cms	
6.	Weight kgs	
7.	Body Fat %	
8.	Visceral Fat %	
9.	Skeletal Muscle %	
10.	To which club you are affiliated?	☐ Nepal Police Club<br>☐ Nepal Armed Police Force
11.	What is your ethnicity?	☐ Brahmin/chhetri<br>☐ Janajati<br>☐ Dalit<br>☐ Terai caste<br>☐ others
12.	What is your educational qualification?	☐ primary (1-5)<br>☐ secondary (6-10)<br>☐ higher secondary (11-12)<br>☐ Bachelors and above
13.	What is your religion?	☐ hindu<br>☐ Boudhist<br>☐ Muslim<br>☐ kirat<br>☐ christian<br>☐ Others
14.	What is your marital status?	☐ Married<br>☐ Unmarried
15.	What is your main occupation?	☐ Job<br>☐ Agriculture<br>☐ Business<br>☐ Unemployed<br>☐ Student

		☐ Others
16.	Family monthly income	....
17.	Which sports do you play?	....
18.	Since how long you have been involved in sports	
19.	your daily training hours	☐ Less than 1 hour<br>☐ to 3 hour<br>☐ Above 3 hours
20.	Do you eat any dietary supplements	☐ yes<br>☐ No
21.	If yes, what is its name?	....<br>..
22.	From where, do you get nutrition related information multi response	☐ Books<br>☐ Articles<br>☐ Social media<br>☐ Coach/trainer<br>☐ Dietitian/nutritionist<br>☐ Health workers<br>☐ Friends<br>☐ Newspaper<br>☐ Others
23.	Have you ever attended classes related to nutrition?	☐ Yes<br>☐ No
24.	Do you check the labelling of food items before purchasing?	☐ Yes<br>☐ No
25.	Do you follow diet plan?	☐ Yes<br>☐ No
26.	If yes, from whom you get it.	…………………………
27.	Is there any difference in food intake during off and on season of sports?	☐ Yes<br>☐ No

Sports Nutrition Knowledge questionnaire				
**SN**	**Statements**	**Agree**	**Disagree**	**Don't know**
1	Learning facts about nutrition is the best way to achieve favorable changes in food habits.			
2	The type of food an athlete eats affects his/her performance.			
3	A sound nutritional practice for athletes is to eat a wide variety of different food types from day to day.			
4	Carbohydrate is the major source of energy followed by fat and protein			
5	Increasing protein in the diet is the main dietary change needed when only muscle gain is desired.			
6	A course in nutrition would be helpful for the athlete.			
7	Skipping meal is advisable if you need to lose weight quickly.			
8	Eggs contain all the essential amino acids needed by the body.			
9	Vegetarian athletes can meet their protein requirements without use of protein supplement.			
10	Milk is a good supplier of calcium for all age groups.			
11	Lack of iron in diet can result in fatigue, injury and illness.			
12	Due to menstruation, females need more iron in their diets than men.			
13	Vitamins provide the body with energy.			
14	Vitamin c tablet should always be taken by athletes.			
15	Vitamins are good source of energy.			
16	Athletes should drink water during activity in order to maintain plasma volume.			
17	Regarding fluid intake during physical activity, recommendations encourage athletes to drink to a plan based on body weight changes during training sessions performed in a similar climate.			
18	Carbonated beverages can negatively affect calcium metabolism.			
19	Dehydration can impair physical performance.			
20	Sports drink are the best way to replace body fluids lost during exercise.			
21	Alcohol's consumption can affect absorption and utilization of nutrients.			
22	Caffeine can increase the risk of dehydration.			
23	Before competition, athletes should consume foods that are high in fat.			
24	Pre event meal should be eaten about 3 to 4 hours before competition.			
25	Consuming carbohydrates during exercise will assist in maintaining blood glucose levels.			
26	All supplements are beneficial to use for athletes.			
27	Supplements label may contain false or misleading information.			
28	The purity and safety of all supplements are tested before sale.			
29	World anti-Doping Agency has banned the use of caffeine and bicarbonate			

	World anti-doping Agency ले caffeine and bicarbonate			
30	Carbohydrate loading is significant method to be practiced by endurance athletes.			

Sports Nutrition Attitude Questionnaire

SN	Statements	Strongly Agree	Agree	Undecided	Disagree	Strongly disagree
1	I intend to eat balanced diet every day					
2	I have a set schedule for meals.					
3	I believe that eating proper diet will improve my physical performance.					
4	I always opt for pre event meal 3 to 4 hours before competition.					
5	Taking more eggs is the only way to build muscles.					
6	I have to take supplements to improve my performance.					
7	Fruits and vegetables are also necessary for me to fulfill my nutrient requirements.					
8	I should consume meat and eggs only to fulfill my protein requirement					
9	I seek food or nutrition related advice from Nutritionist or Dietitian.					
10	When considering eating any food, I care what my coach thinks I should do					
11	Balanced diet will help me to be a successful player.					
12	It is my duty to read food labels before consuming it					
13	I have to skip meals to lose my weight					
14	I feel that I cannot perform well when I am dehydrated					
15	Before the competition I should eat heavy meal with high fat and protein					
16	It Is my responsibility to know items that has been banned by World Anti-Doping Agency. World Anti-Doping Agency					

Sports Nutrition Practice Questionnaire

SN	Statements	Yes	No
1	I have set a schedule for meals		
2	I check labelling of food before purchasing or consuming		
3	I always have someone to ask about what should I eat		
4	I ask Nutritionist or Dietitian for nutrition advice.		
5	I eat at least four meals a day		

6	I use supplements to improve my performance		
7	I have been using supplements under the guidance of medical personnel or Nutritionist		
8	I consume fruits and vegetables daily		
9	During exercise/training session, I drink electrolytic beverages		
10	I eat pre-event meal before 3 to 4hours of competition		
11	I eat food high in simple carbohydrates and protein before competition		
12	I consume fluids containing carbohydrate during competition		
13	I consume food high in protein, carbohydrate and protein after competition		
14	I avoid deep fried, oily and spicy foods		

24-hour recall

What you eat usually throughout the day? (From midnight to midnight the previous day during their training

**Time**	**Food items**	**Ingredients**	**Quantity**	**Household measurement**
Pre work out meal				
Breakfast				
Post workout meal				
Launch				
Mid-day snack				
Dinner				
